# Healthcare utilization following hip fractures based on social vulnerability status in the US: an analysis of 2016–2020 nationwide readmissions data

**DOI:** 10.1007/s00402-026-06322-3

**Published:** 2026-05-02

**Authors:** Ria Tilve, Guangjin Zhou, Shujaa T. Khan, Siran M. Koroukian, Matthew Deren, Nicolas S. Piuzzi

**Affiliations:** 1 Case Western Reserve University, Cleveland, USA; 2 Department of Population and Quantitative Health Sciences, Case Western Reserve University, Cleveland, USA; 3 Department of Orthopedic Surgery, Cleveland Clinic, Cleveland, USA

**Keywords:** Social determinants of health, Healthcare utilization, Hip fracture, Complications

## Abstract

**Introduction:**

Social vulnerability (SV) influences rehabilitation and postoperative care for patients with hip fracture. However, most previous work relies on area-level measures that overlook interindividual variation. The recent adoption of ICD-10 Z-codes allows clinical identification of patient-level SV and may offer a better understanding of its impact. This study aimed to evaluate healthcare utilization, including readmissions, discharge disposition, and length of stay (LOS) in surgically treated hip fracture patients with and without clinically acknowledged SV.

**Methods:**

Adults surgically treated for hip fracture between 2016 and 2020 were included from the Nationwide Readmissions Database. SV was defined as having at least one documented relevant ICD-10 Z-code. Primary outcome measures included complications, LOS, discharge disposition, and 30- and 90-day readmissions, stratified by SV and evaluated using chi-square analyses. Multivariable logistic regression assessed long LOS (≥ 5 days) and discharge to home, adjusting for age, insurance/income status, and substance use.

**Results:**

Patients with SV were younger (35.6% with SV vs. 50.1% without SV were 81+), had a lower median household income (38.8% with SV vs. 25.7% without SV were in the lowest quartile), and were more often insured by Medicaid (19.3% vs. 3.8%). Alcohol/drug use disorders were significantly more prevalent in patients with SV (18.5% vs. 4.5%). SV was associated with 47% higher odds for long LOS (1.47, 1.41–1.54) and 23% higher odds for discharge to home (1.23, 1.16–1.30) but comparable 90-day readmissions (21.2% vs. 19.8%).

**Conclusion:**

Among surgically treated hip fracture patients, SV was associated with higher odds of long LOS and discharge to home but no meaningful difference in readmissions. The small number of patients with clinically documented SV highlights the limited reporting by healthcare workers. This analysis of a nationwide all-payer database highlights the need to identify these higher risk patients and implement appropriate care pathways to reduce healthcare utilization.

**Supplementary Information:**

The online version contains supplementary material available at 10.1007/s00402-026-06322-3.

## Introduction

Hip fracture is one of the most common orthopedic conditions requiring hospitalization, affecting over 350,000 people and creating an economic burden of over $17 billion each year in the United States [1]. Due to the increasing incidence and burden, reducing hip fractures has been included in the US Department of Health’s Healthy People 2030 [2]. Furthermore, hip fractures significantly increase mortality, with 30% of people suffering a hip fracture dying within the next year [3]. Over 90% of people presenting with a hip fracture are over 65 years old commonly have pre-existing conditions, making adequate post-operative care imperative to improving health outcomes [4, 5]. Despite improvements in treatment protocols, complications are prevalent and estimated to affect at least 20% of patients [4]. In fact, early readmissions after hip fracture have been increasing over the last decade, suggesting that non-medical risk factors likely play an important role in successful recovery [6]. 

Hip fractures significantly impact quality of life even after recovery, as many patients suffer marked deterioration in functional capacity [7]. As a result, rehabilitation from a hip fracture requires both physical and psychological support [8, 9]. Access to adequate post-injury care is closely dependent on factors that determine the systemic and structural conditions of patients’ lives [10–12]. Social vulnerability (SV), defined here as the presence of at least one documented health-related social need (such as food insecurity), is associated with increased susceptibility and negative impacts on patients’ health and has been shown to affect outcomes in patients with hip fracture [13–16]. 

Previous studies evaluating SV in hip fracture outcomes have data from single institutions, and those using multi-institutional data assessed social needs using race, socioeconomic status, or other characteristics as a proxy for determining the presence of SV [17–22]. Such studies assessing specific social needs, like income or educational attainment, used area-level measures like zip codes or census data, which may not always account for variability within a population [23–26]. 

Starting in 2015, “Z-codes” were introduced to the ICD-10 classification system to help identify health-related social needs that can influence a patient’s health status [27]. These Z-codes identify nonmedical factors across several domains, including employment, family, housing, psychosocial, socioeconomic status, and dependence [28]. Accordingly, SV defined by Z-codes reflects clinically acknowledged social needs rather than true population prevalence. When documented, Z-codes allow for objective identification of SV and can be used to gain a more comprehensive understanding of the impact of SV on health outcomes, like recovery after a hip fracture, at an individual-level. While previous studies have shown the link between individual social needs and hip fracture outcomes, a broader understanding of this association at the national level is needed to better address these disparities in health outcomes [29–31]. In this study, a large, national all-payer database was used to determine the prevalence of documented SV in hip fracture patients, compare comorbidities and complications among patients with and without documented SV, and evaluate the association of documented SV with length of stay (LOS), discharge disposition, and hospital readmissions.

## Methods

### Data source

The Nationwide Readmissions Database (NRD) was queried for all surgically treated hip fracture patients from 2016 to 2020 [32]. The NRD is an administrative database from the Healthcare Cost and Utilization Project maintained by the Agency for Healthcare Research and Quality. The NRD is the largest public database containing national data on readmissions in the US regardless of insurance status [33]. The NRD contains information from 21 to 31 states and 2,350-2,550 hospitals, is geographically dispersed, and accounts for between 57 and 62% of the US population and 56–61% of all US hospitalizations [33]. These data were weighted to obtain nationally representative estimates of over 30 million discharges annually [33]. This study was deemed exempt by the Institutional Review Board.

## Study population

ICD-10 codes were used to identify patients with hip fractures who underwent surgical treatment by total hip arthroplasty, hemiarthroplasty, or internal fixation based on previously published studies (Online Resource Table S1) [34–36]. Patients who died during the index visit or patients with missing data for the unique patient identifier and LOS were excluded in analyses of hospital readmissions.

### Outcomes

Complications were derived using data at the index visit and subsequent admissions. Complications were defined using either ICD-10 diagnosis or procedure codes and coded as binary variables. These included dialysis, transfusion, stroke, pneumonia, cardiac and gastrointestinal complications, pulmonary embolism/deep vein thrombosis, delirium, and infection. LOS describing the number of inpatient days during the index visit was dichotomized using a cutoff of 5 days reflective of the average hospital LOS to calculate odds ratios (long LOS was 5 + days, short LOS was < 5 days) [37]. Discharge disposition was described with categories including discharge to home/routine discharge, transfer to short-term facility or discharge to skilled nursing facility (SNF), or discharge to others. Discharge disposition was also dichotomized into discharge to home or not discharge to home, which included every other discharge category, for regression analysis.

Readmissions were defined as any admission after being discharged alive from the index admission during which the surgery was performed. Readmissions were categorized as 30-day (within 30 days) or 90-days (within 90 days) after the day of discharge from the index visit. NRD data allow for tracing a patient within the same calendar year, so the study population was limited to hip fracture patients with an index visit from January through November or January through September of each year to ensure complete 30-day or 90-day follow up, respectively.

### Independent variables

SV status was the main independent variable and defined using ICD-10 Z-codes listed at the index visit or subsequent visits during any year. Based on previous studies, ICD-10 Z-codes for health-related social needs were classified into five major domains of SV: employment, family, housing, psychosocial needs, and socioeconomic status (SES) (Online Resource Table S2) [38]. If a subsequent visit did not list any Z-codes, the patient was still included in the initial social vulnerability domain(s) identified. Patients with multiple Z-codes were counted in each associated domain and only counted once in any particular domain.

Patient demographics included age, sex (male/female), procedure type, admission year, median household income (quartiles), insurance type (Medicaid, Medicare, private, other, uninsured), hospital type (metropolitan teaching, metropolitan non-teaching, non-metropolitan), and hospital ownership (government, private, private investor-owned). Patient comorbidities were identified using ICD-10 codes and Elixhauser Comorbidity Software Refined for ICD-10 as defined by HCUP in the NRD [33]. 

### Statistical analysis

 Patients were stratified by SV status and associations with outcomes (complications, LOS, discharge disposition, 30- and 90- day readmissions) were evaluated using chi-square analyses. Multivariable logistic regression analysis was conducted to assess the associations between SV and each of LOS and discharge disposition. Given the similar readmission rates by SV status, regression models for readmissions were not analyzed but are included as supplemental information (Online Resource Table S3). Statistical significance was defined as *p* < 0.05. The patient cohort was created and data analysis was conducted using Linux based SAS Software 9.4.

## Results

**Fig. 1:**
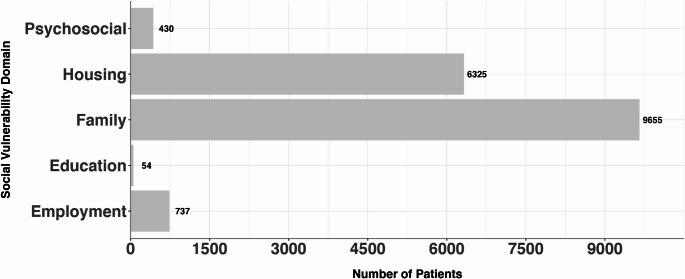
Distribution of social vulnerability domains in patient population: Family has the most patients with 9655 patients, followed by Housing (6325), Employment (737), Psychosocial (430), and lastly, Education with 54 patients

Of the 1,555,988 patients undergoing surgical management for hip fracture, 16,862 patients.

(1.08%) had SV (Fig. 1). The most frequently documented domains of SV were family-related (57.5%), followed by housing (37.5%), psychosocial (2.5%), and employment (4.4%). Education (0.3%) needs were least commonly documented (Fig. 1).

Patients with SV were younger (35.6% with SV vs. 50.1% with no SV were 81+), male (45.6% vs. 33.4%) and in the two lowest median household income quartiles than patients without SV (Table 1). More patients with SV were insured by Medicaid (19.3% vs. 3.8%) or were uninsured (4.5% vs. 1.2%), while more patients without SV were using Medicare (82.9% vs. 65.0%) or private insurance (9.2% vs. 6.4%) (Table 1) Proportionally more patients with SV were treated with internal fixation (69.3% vs. 63.6%), while proportionally more patients without a SV were treated with total hip arthroplasty (8.1% vs. 6.4%) or hemiarthroplasty (29.6% vs. 25.5%) (Table 1). The number of patients with SV surgically treated for a hip fracture nearly doubled over the specified time period (2,952 in 2016 vs. 4,581 in 2020), while the number of patients without a SV increased only marginally (299,997 in 2016 vs. 309,325 in 2020) (Table 1). All of these differences in patient characteristics were significant (*p* < 0.05).

**Table 1:** Patient population stratified by social vulnerability status

	No Social Vulnerability (*N* = 1,539,126)		Social Vulnerability (*N* = 16,862)	
Variable	*N*	(%)	*N*	(%)
Treatment				
Total hip arthroplasty	124,320	8.1%	1,082	6.4%
Hemiarthroplasty	456,206	29.6%	4,300	25.5%
Internal Fixation	979,450	63.6%	11,686	69.3%
Age (Years)				
40 and under	38,796	2.5%	942	5.6%
41 to 50	25,587	1.7%	853	5.1%
51 to 60	91,037	5.9%	2,740	16.2%
61 to 70	222,079	14.4%	3,212	19.0%
71 to 80	390,277	25.4%	3,113	18.5%
81+	771,349	50.1%	6,002	35.6%
Sex				
Male	513,381	33.4%	7,697	45.6%
Female	1,025,746	66.6%	9,166	54.4%
Median Household Income				
1 st Quartile (Lowest)	396,154	25.7%	6,548	38.8%
2nd Quartile	431,577	28.0%	4,158	24.7%
3rd Quartile	380,757	24.7%	3,180	18.9%
4th Quartile (Highest)	311,722	20.3%	2,580	15.3%
Insurance				
Medicaid	58,974	3.8%	3,250	19.3%
Medicare	1,276,206	82.9%	10,963	65.0%
Other	43,250	2.8%	796	4.7%
Private	142,064	9.2%	1,087	6.4%
Uninsured (Self)	18,632	1.2%	766	4.5%
Teaching status of hospital*				
Metropolitan non-teaching	380,849	24.7%	3,615	21.4%
Metropolitan teaching	978,582	63.6%	11,466	68.0%
Non-metropolitan hospital	179,696	11.7%	1,781	10.6%
Index Admission Year				
2016	299,997	19.5%	2,952	17.5%
2017	303,707	19.7%	2,787	16.5%
2018	308,440	20.0%	2,998	17.8%
2019	317,658	20.6%	3,544	21.0%
2020	309,325	20.1%	4,581	27.2%
Hospital Ownership				
Government	165,982	10.8%	2,883	17.1%
Private	1,158,696	75.3%	11,224	66.6%
Private, invest-own	214,448	13.9%	2,755	16.3%

* indicates *p* = 0.0011, all comparisons were significant at *p* < 0.0001

With regard to comorbidities, more patients with than without SV had a history of alcohol use disorders (18.5% vs. 4.5), substance use disorders (11.5% vs. 1.7%), depression (18.7% vs. 16.2%), and psychosis (9.2% vs. 3.2%) (Table 2). Liver disease (6.2% vs. 2.6%) and significant weight loss (12.6% vs. 8.7%) were also more common in patients with SV (Table 2). Conversely, more patients with no SV had a history of arrhythmias (23.9% vs. 18.7%), congestive heart failure (16.3% vs. 11.5%), diabetes mellitus (uncomplicated 10.1% vs. 6.7%, complicated 13.4% vs. 11.1%), hypertension (72.2% vs. 63.6%), and renal failure (18.7% vs. 12.0%) (Table 2). All of these differences in patient comorbidity profiles were significant (*p* < 0.05).

**Table 2:** Patient comorbidities stratified by social vulnerability status

	No social vulnerability (*N* = 1,539,126)		Social vulnerability (*N* = 16,862)	
Comorbidities	*N*	(%)	*N*	(%)
AIDS	1,462	0.1%	66	0.4%
Alcohol use disorder	69,949	4.5%	3,124	18.5%
Arrhythmia	368,013	23.9%	3,154	18.7%
Arthritis	61,942	4.0%	563	3.3%
Deficiency anemia	357,951	23.3%	4,492	26.6%
Blood loss anemia	29,423	1.9%	451	2.7%
Congestive heart failure	251,030	16.3%	1,938	11.5%
Chronic lung disease	341,593	22.2%	4,144	24.6%
Coagulopathy	72,296	4.7%	951	5.6%
Depression	249,985	16.2%	3,145	18.7%
Diabetes mellitus - uncomplicated	155,028	10.1%	1,136	6.7%
Diabetes mellitus - complicated	205,544	13.4%	1,868	11.1%
Substance use disorder	25,545	1.7%	1,938	11.5%
Hypertension	1,110,985	72.2%	10,717	63.6%
Hypothyroidism	321,164	20.9%	2,718	16.1%
Electrolyte Imbalance	482,406	31.3%	6,020	35.7%
Lymphoma	10,822	0.7%	72	0.4%
Liver disease	39,678	2.6%	1,041	6.2%
Metastatic Cancer	19,121	1.2%	137	0.8%
Neurological disease	288,900	18.8%	2,628	15.6%
Obesity	100,235	6.5%	1,002	5.9%
Paralysis^†^	59,052	3.8%	588	3.5%
Peripheral vascular disease	116,767	7.6%	1,136	6.7%
Psychosis	48,534	3.2%	1,559	9.2%
Pulmonary circulation disorders^†^	13,667	0.9%	148	0.9%
Renal failure	287,321	18.7%	2,027	12.0%
Tumor^†^	30,783	2.0%	296	1.8%
Ulcer^†^	9779	0.6%	88	0.5%
Valvular disease	160,674	10.4%	1,422	8.4%
Weight loss	133,225	8.7%	2,130	12.6%

† indicates comparison was not significant, all other comparisons were significant at *p* < 0.05

The most common complications in both patient groups were non-mechanical complications from the index procedure, and nearly half experienced acute posthemorrhagic anemia (48.6% in both groups) (Table 3 A). For both groups of patients, cardiac-related complications appeared to be the most common, with a significantly smaller percentage of patients with SV affected compared to patients without SV (31.0% vs. 38.4%, *p* < 0.05) (Table 3B).

**Table 3:** Complications from index procedure stratified by social vulnerability status

(A) Specific Complications by Category					
	No Social Vulnerability (*N* = 1,539,126)		Social Vulnerability (*N* = 16,862)		
Category	*N*	(%)	*N*	(%)	*P*-Value
Mechanical Complications	11,873	0.8%	116	0.7%	0.3695
Mechanical loosening	3,262	0.2%	22	0.1%	0.0825
Dislocation of prosthetic joint	3,552	0.2%	42	0.2%	0.7102
Prosthetic joint implant failure	566	0.0%	*	*	0.2415
Periprosthetic fracture	3,586	0.2%	40	0.2%	0.9266
Periprosthetic osteolysis	437	0.0%	*	*	0.7176
Articular bearing surface wear	325	0.0%	0	0.0%	–
Unspecified/other mechanical	1,140	0.1%	11	0.1%	0.6275
Non-mechanical Complications	1,123,236	73.0%	11,689	69.3%	< 0.0001
Postoperative shock	3,116	0.1%	*	*	0.0059
Hemorrhage/hematoma/seroma	4,878	0.3%	68	0.4%	0.1601
Disruption of wound	1,504	0.1%	25	0.1%	0.1807
Acute posthemorrhagic anemia	748,639	48.6%	8,199	48.6%	0.9777
Pulmonary embolism and infarction	131,821	8.6%	1,243	7.4%	0.0009
Lower extremity deep vein thrombosis	12,940	0.8%	217	1.3%	< 0.0001
Pulmonary insufficiency	11,041	0.7%	119	0.7%	0.9145
Infection-Related Complications	208,649	13.6%	2,076	12.3%	0.0016
Postoperative infection	3,472	0.2%	67	0.4%	0.0019
Infection/inflammatory reaction	1,775	0.1%	27	0.2%	0.2307
Urinary tract infection	204,378	13.3%	1,998	11.8%	0.0003
Cellulitis/abscess	0	0.0%	0	0.0%	–
Mortality	26,931	1.7%	181	1.1%	< 0.0001

(B) Complications by General Organ System					
	No Social Vulnerability (*N* = 1,539,126)		Social Vulnerability (*N* = 16,862)		
Organ System	*N*	(%)	*N*	(%)	*P*-Value
Central nervous system	35,148	2.3%	346	2.1%	0.1528
Cardiac system	591,509	38.4%	5,227	31.0%	< 0.0001
Peripheral vascular system	766	0.0%	*	*	0.1093
Respiratory system	62,635	4.1%	630	3.7%	0.1394
Gastrointestinal system	15,329	1.0%	222	1.3%	0.0034
Genitourinary system	4,393	0.3%	38	0.2%	0.2697

* denotes cells with frequency < 11 were suppressed due to requirements of HCUP data use agreement

Comparison of unadjusted results reveals that a higher percentage of patients without SV had long LOS compared to patients with social vulnerability (45.5% vs. 33.0%) (Table 4). 30-day readmissions (19.7%) and 90-day readmissions (21.2%) were slightly more common among patients with SV (Table 4). Infection-related conditions, including sepsis, urinary tract infection, acute kidney failure, and pneumonia, were the most common primary diagnoses for readmissions for both groups of patients (Online Resource Table S4). Patients with SV also had a higher prevalence of readmission related to alcohol dependence and cellulitis, while patients without SV had a higher prevalence of readmissions related to aspiration pneumonia and pulmonary embolism (Online Resource Table S4).

**Table 4:** Healthcare utilization stratified by social vulnerability status: Length of stay, discharge disposition, 30-day readmissions, and 90-day readmissions

	No Social Vulnerability		Social Vulnerability		
	*N*	(%)	*N*	(%)	*P*-Value
Binary Length of Stay	1,539,127		16,862		< 0.0001
Long (5 + days)	700,530	45.5%	5,563	33.0%	
Short (< 5 days)	838,597	54.5%	11,299	67.0%	
Total 30-day Readmissions	**260**,**006**	**18.9%**	**2**,**970**	**19.7%**	**0.0147**
1	206,431	79.4%	2,256	76.0%	0.0011
2	42,388	16.3%	542	18.3%	
3+	11,187	4.3%	172	5.8%	
Total 90-day Readmissions	**221**,**152**	**19.8%**	**2**,**553**	**21.2%**	**0.0001**
1	173,714	78.6%	1,933	75.7%	0.0122
2	37,131	16.8%	464	18.2%	
3+	10,267	4.6%	156	6.1%	
Discharge Destination	**1**,**538**,**611**		**16**,**836**		**< 0.0001**
Home Health Care	261,507	17.0%	2,922	17.3%	
Home	161,754	10.5%	3,358	19.9%	
Other	32,102	2.1%	457	2.7%	
Transfer to SNF	1,072,492	69.7%	9,925	58.9%	
Transfer to short term facility	10,756	0.7%	174	1.0%	

Discharge to home (19.9% vs. 10.5%) was significantly more common for patients with SV, whereas discharge to a SNF was significantly more common for patients without SV (69.7% vs. 58.9%) (Table 4). Most of the patients with SV discharged to home had private insurance (40.4%), while most patients not discharged to home had Medicare (75.1%) (Table 5). Most patients without SV had Medicare, regardless if they were discharged to home (48.1%) or not (87.0%) (Table 5). All of these differences in healthcare utilization were significantly different (*p* < 0.05).

**Table 5:** Discharge to home by insurance type for patients with and without documented social vulnerability

	Discharged to Home		Not Discharged to Home		
	*N*	(%)	*N*	(%)	*P*-Value
Social Vulnerability	3,358		13,504		< 0.0001
Private	1,355	40.4%	1,894	14.0%	
Medicare	822	24.5%	10,141	75.1%	
Medicaid	371	11.0%	426	3.2%	
Other	275	8.2%	812	6.0%	
Uninsured	535	15.9%	231	1.7%	
No Social Vulnerability	**161**,**754**		**1**,**377**,**372**		**< 0.0001**
Private	20,839	12.9%	38,135	2.8%	
Medicare	77,758	48.1%	1,198,448	87.0%	
Medicaid	12,423	7.7%	30,827	2.2%	
Other	40,071	24.8%	101,993	7.4%	
Uninsured	10,662	6.6%	7,970	0.6%	

After adjusting for sex, age, income, insurance, and substance use disorders, patients with SV were more likely to have a longer LOS (adjusted OR 1.47, 95% CI: 1.41–1.54). Age was also significantly related to discharge disposition and LOS, with 81 years and older having significantly lower odds of being discharged to home (adjusted OR 0.05, 95% CI: 0.05–0.06) and significantly higher odds of having longer LOS (adjusted OR 2.03, 95% CI: 1.96–2.01) compared to patients 40 years of age and younger (Table 6). Adjusting for the same variables also showed that patients with SV were more likely to be discharged to home (adjusted OR 1.23, 95% CI: 1.16–1.30) (Table 6). Male (vs. female) sex, lower (vs. higher) median household income quartile, and uninsured, Medicaid, and other (vs. private) insurance all had higher odds of being discharged to home and higher odds of having a long LOS (Table 6). Patients with no alcohol or substance use disorders had higher odds of being discharged to home and lower odds of long LOS (Table 6).

**Table 6:** Multivariable logistic regression models for discharge to home and length of stay

	Discharge to Home	Long Length of Stay (5 + days)
Variable	Odds Ratio (95% CI)	Odds Ratio (95% CI)
Social Vulnerability = Yes	1.23 (1.16–1.30)	1.47 (1.41–1.54)
Sex = Male	1.12 (1.10–1.14)	1.24 (1.23–1.25)
Age (Years)		
Under 40	Reference	Reference
41 to 50	0.49 (0.46–0.51)	1.11 (1.06–1.16)
51 to 60	0.30 (0.29–0.31)	1.25 (1.21–1.30)
61 to 70	0.21 (0.20–0.22)	1.49 (1.44–1.54)
71 to 80	0.12 (0.11–0.12)	1.75 (1.70–1.81)
81 and above	0.05 (0.05–0.06)	2.03 (1.96–2.09)
Median Household Income		
1 st Quartile (Lowest)	1.19 (1.17–1.22)	1.37 (1.36–1.39)
2nd Quartile	1.16 (1.13–1.19)	1.17 (1.16–1.19)
3rd Quartile	1.11 (1.08–1.13)	1.10 (1.09–1.11)
4th Quartile (Highest)	Reference	Reference
Insurance		
Private	Reference	Reference
Medicare	0.44 (0.43–0.45)	1.07 (1.05–1.08)
Medicaid	1.01 (0.98–1.04)	1.56 (1.52–1.60)
Other	1.17 (1.13–1.21)	1.14 (1.10–1.17)
Uninsured	2.83 (2.70–2.97)	1.04 (1.00–1.09.00.09)
Alcohol Use Disorder = Yes	0.80 (0.78–0.82)	1.41 (1.38–1.44)
Drug Use Disorder = Yes	0.89 (0.85–0.93)	1.41 (1.37–1.46)

## Discussion

Nonmedical risk factors associated with SV can have significant impacts on patient care, but individual-level SV is difficult to identify. The recent introduction of ICD-10 Z-codes has allowed healthcare providers to clinically acknowledge patient SV. Applying these Z-codes to a national all-payer database would provide a broader understanding of SV and its relation to healthcare utilization, particularly in patients with hip fractures. Given the large numbers, interpretations were based on clinical meaningfulness of the differences, rather than the statistical significance. Among patients surgically treated for hip fractures, SV was associated with higher odds of long LOS and discharge to home, but differences in readmission rates were not clinically meaningful. This analysis highlights the need to promote standardized SV documentation to help identify higher-risk patients and reduce healthcare utilization patterns to optimize patient outcomes.

In this population, patients with SV were generally younger, male, and in lower median household income quartiles, all characteristics commonly associated with high-impact trauma requiring more immediate attention [39–42]. The comorbidity profile of patients with SV was also significantly different, showing increased rates of alcohol and drug use disorders, depression, and psychosis. Previous studies have also found that factors related to social relationships, including family dependence and interpersonal conflicts, are associated with having a history of these psychosocial comorbidities [43, 44]. The varying comorbidity profiles could also be a result of the different age and insurance type distribution among the patient groups. Arrhythmias, congestive heart failure, diabetes, hypertension, and renal failure were more common for patients without SV and are known to become much more common with increasing age [45–47]. Furthermore, mood and psychosocial conditions are the most common reasons for readmissions for patients with Medicaid or no insurance, while maintenance care and cardiopulmonary conditions are more common reasons for readmission among patients with Medicare or private insurance [48]. These close associations between chronic health conditions, age, and insurance type have been observed in previous studies on hip fracture outcomes [35, 49]. Differences in comorbidity profiles of these patients may also explain the increased variability in LOS, as patients with SV had lower unadjusted odds of prolonged LOS but higher adjusted odds of prolonged LOS [50]. Adjusted regression models took into account age, income level, insurance status, and alcohol and drug use, so this reversal in odds suggests LOS is highly influenced by patient-specific characteristics, including weight-bearing status, procedural details, and existing health conditions [36, 51–53]. 

Notably, the number of patients with SV with hip fractures nearly doubled over this period, while the proportion of patients without SV remained stable, potentially indicating increased access to care for populations with increased SV. However, other studies have shown that significant disparities in treatment still exist, and it is more likely that Z-codes have been increasingly adopted by providers since its introduction in 2015, resulting in more frequent identification in later years [20]. The distribution of mechanical complications was also similar between groups, suggesting both groups received the same quality of care regardless of SV. Previous work has produced contradictory results, with some analyses finding that lower socioeconomic status is associated with increased mortality risk after hip fractures and others suggesting no association [20, 54]. Further study will be required to clarify the role of how SV status might affect complication risk for hip fractures over time.

Another critical feature of hip fracture outcomes is discharge disposition. The financial strain of obtaining healthcare might explain why patients with SV had higher odds of discharge to home compared to patients without SV [55]. Because post-acute care increases overall healthcare costs, access to these services is often limited to patients with the financial resources available to overcome cost-related barriers or to medically complex patients for whom such care is necessary [20, 36, 55]. A high proportion of patients with SV were in the lowest income quartile, likely reflecting the higher rates of Medicaid or uninsured status, longer LOS, and an greater odds of discharge to home. These findings can highlight Medicaid’s role as an essential safety net for these patients, as insurance status and income level are key predictors of access to care [55]. 

Age also affects discharge disposition, with increasing age observed to have decreasing odds of discharge to home [55]. Among patients with Medicare, those with SV were nearly three times less likely to be discharged to home, while patients without SV were about half as likely to be discharged to home. Medicare insurance, and by association older age, appears to have lower rates of discharge to home despite differences in SV. As a result, the younger age distribution among patients with SV might explain the overall higher rate of discharge to home in this population. Previous work has shown that patients discharged to home are also consequently more likely to experience readmissions, which may explain why patients with SV in this cohort had somewhat higher rates of 90-day readmissions [56]. 

While previous studies have reported on the importance of social factors on hip fracture outcomes, most relied on data from specific insurance populations, area-level data, or subjective survey responses, limiting their application to broader populations [3, 8, 22, 26]. This analysis of a national all-payer database allowed for a better understanding of social factors across insurance types and between individuals. The use of Z-codes provided a novel and objective way to identify SV present in patients but limited the population to cases when SV was clinically acknowledged, producing selection bias. Patients with less severe presentations of these SV might not have been identified and documented, making these findings less generalizable. Patients with less severe presentations of these SV might not have been identified and documented. Z-codes are also new with limited guidance and subjective definitions, so healthcare workers might not be aware of the if, when and how it is appropriate to use such definitive diagnoses, leading to the extremely small number of patients identified with SV. Furthermore, only patients seen at healthcare facilities included in the HCUP data were included in this dataset. Because SV status is tied to access to care, it is possible that the most severely affected patients were not able to obtain care and thus not represented [10]. An important variable not included in this study was race/ethnicity, as this information was not available in the NRD. Such information could also impact both SV status and hip fracture outcomes, making it an important confounder that needs further study among these relationships [1, 20]. 

## Conclusions

Through utilization of novel ICD-10 Z-codes to identify SV in surgically treated hip fractures patients, clinically acknowledged SV was found to be associated with 47% higher odds of increased LOS and 23% higher odds of discharge to home. The small number of patients with SV identified highlights the limited adoption of Z-codes by healthcare workers. Given the relationships between SV and patient outcomes, standardizing Z-code use among healthcare workers could help with identifying more high-risk patients and implementing appropriate care protocols to reduce healthcare utilization and improve outcomes.

## Supplementary Information

Below is the link to the electronic supplementary material.

Supplementary Material 1

## Data Availability

The data that support the findings of this study are available from the authors but restrictions apply to the availability of these data, which were used under a data use agreement with the Healthcare Cost and Utilization Project (HCUP) for the current study. Data are available from the authors upon reasonable request and with permission from HCUP.
