# Supplementary material for: Healthcare utilization following hip fractures based on social vulnerability status in the US: an analysis of 2016–2020 nationwide readmissions data
Source: Arch Orthop Trauma Surg. 2026 May 2;146(1):172. doi: 10.1007/s00402-026-06322-3 (PMC13135577; doi:10.1007/s00402-026-06322-3)
Supplement: Supplementary file 1 — Supplementary Material 1 [file 402_2026_6322_MOESM1_ESM.pdf]

Supplementary Information

Title: Healthcare Utilization following Hip Fractures based on Social Vulnerability Status in the US: An Analysis of 2016-2020 Nationwide Readmissions Data

Authors:
Ria Tilve, MPH[1]
Guangjin Zhou, PhD[2]
Shujaa T. Khan, MD[3]
Ignacio Pasqualini, MD[3]
Siran M. Koroukian, PhD[2]
Matthew Deren, MD[3]
Nicolas S. Piuzzi, MD[3]

[1] Case Western Reserve University School of Medicine, Cleveland, Ohio
[2] Department of Population and Quantitative Health Sciences, Case Western Reserve University, Cleveland, Ohio
[3] Cleveland Clinic Foundation Department of Orthopedic Surgery, Cleveland, OH, USA 44195

Corresponding Author:
Nicolas Piuzzi, MD
Cleveland Clinic
Orthopedic and Rheumatology Institute
9500 Euclid Ave, A41
Cleveland, OH 44195
Email: piuzzin@ccf.org
Tel: 216-636-1136
Fax: 216-445-6255

Table S1. ICD-10 Codes used to define patient population.

**Diagnosis Codes**	S72.0x - Fracture of head and neck of femur<br>S72.1x - Pertrochanteric fracture<br>S72.2x - Subtrochanteric fracture of femur
**Treatments**	**Total Hip Arthroplasty**<br>0SR9x, 0SRBx<br><br>**Hemiarthroplasty**<br>0SRRx, 0SRSx, 0QR6x, 0QR7x<br><br>**Internal Fixation**<br>0QQ6x, 0QQ7x, 0QS6x, 0QS7x, 0QH6x, 0QH7x, 0SQ9x, 0SQBx

Table S2. ICD-10 Codes used to identify social vulnerabilities.

**Social Vulnerability Domain**	**ICD-10 Codes**
Family	Z60.0, Z60.2, Z60.3, Z60.4, Z60.5, Z60.8, Z60.9, Z62.0, Z62.1, Z62.21, Z62.22, Z62.29, Z62.3, Z62.6, Z62.810, Z62.811, Z62.812, Z62.813, Z62.9, Z63.0, Z63.1, Z63.4, Z63.5, Z63.8, Z63.9, Z63.31, Z63.32, Z63.71, Z63.72, Z63.79
Housing	Z590, Z591, Z592, Z593, Z594, Z595, Z596, Z597, Z598, Z599
Psychosocial	Z640, Z641, Z644, Z650, Z651, Z652, Z653, Z654, Z658, Z659
Education	Z550, Z551, Z552, Z553, Z554, Z558, Z559
Employment	Z560, Z561, Z562, Z563, Z564, Z565, Z566, Z569, Z570, Z571, Z572, Z574, Z575, Z576, Z577, Z578, Z579 Z5681, Z5682, Z5689, Z5731, Z5739

Table S3. Multivariable logistic regression models for 30-day and 90-day readmissions.

	30-day Readmissions	90-day Readmissions
**Variable**	**Odds Ratio (95% CI)**	**Odds Ratio (95% CI)**
**Social Vulnerability = Yes**	1.02 (0.95-1.09)	1.09 (1.04-1.15)
**Sex = Male**	1.43 (1.41-1.45)	1.39 (1.38-1.41)
**Age (Years)**		
Under 40	Reference	Reference
41 to 50	1.52 (1.39-1.66)	1.68 (1.57-1.80)
51 to 60	1.84 (1.71-1.97)	2.07 (1.96-2.19)
61 to 70	1.84 (1.72-1.98)	2.01 (1.91-2.13)
71 to 80	2.07 (1.93-2.22)	2.11 (2.00-2.23)
81 and above	2.40 (2.24-2.57)	2.33 (2.20-2.46)
**Median Household Income**		
1st Quartile (Lowest)	1.15 (1.13-1.17)	1.17 (1.15-1.19)
2nd Quartile	1.08 (1.06-1.10)	1.08 (1.06-1.10)
3rd Quartile	1.05 (1.02-1.07)	1.06 (1.04-1.08)
4th Quartile (Highest)	Reference	Reference
**Insurance**		
Private	Reference	Reference
Medicare	1.44 (1.40-1.49)	1.61 (1.57-1.65)
Medicaid	1.45 (1.39-1.52)	1.56 (1.50-1.62)
Other	0.93 (0.87-0.98)	0.89 (0.85-0.94)
Uninsured	0.89 (0.81-0.97)	0.89 (0.83-0.95)
**Alcohol Use Disorder = No**	1.03 (0.99-1.06)	1.08 (1.05-1.11)
**Drug Use Disorder = No**	1.30 (1.24-1.37)	1.47 (1.41-1.53)

Table S4. Top 10 Primary Diagnoses for (a) 30-day and (b) 90-day readmissions, stratified by social vulnerability status.

(A)

No Social Vulnerability (N = 260,006)		Social Vulnerability (N = 15,056)	
**Diagnosis**	**Weighted Frequency (%)**	**Diagnosis**	**Weighted Frequency (%)**
Sepsis, unspecified organism	26495 (10.20%)	Sepsis, unspecified organism	220 (7.41%)
Urinary tract infection, site not specified	7450 (2.87%)	Urinary tract infection, site not specified	56 (1.90%)
Acute renal failure, unspecified	6356 (2.45%)	Acute renal failure, unspecified	51 (1.73%)
Pneumonia, unspecified organism	6142 (2.36%)	Infection following a procedure	44 (1.49%)
Hypertensive heart and chronic kidney disease with heart failure and stage 1 through stage 4 chronic kidney disease, or unspecified chronic kidney disease	4901 (1.89%)	Pneumonia, unspecified organism	51 (1.72%)
Hypertensive heart disease with heart failure	4599 (1.77%)	Hypertensive heart disease with heart failure	51 (1.72%)
Pneumonitis due to inhalation of food and vomit	4297 (1.65%)	Dislocation of internal left hip prosthesis, initial encounter	30 (1.02%)
Dislocation of internal left hip prosthesis, initial encounter	3821 (1.47%)	Chronic obstructive pulmonary disease with (acute) exacerbation	31 (1.05%)
Other pulmonary embolism without acute cor pulmonale	3442 (1.32%)	Cellulitis of left lower limb	31 (1.04%)
Dislocation of internal right hip prosthesis, initial encounter	3231 (1.24%)	Alcohol dependence with withdrawal, unspecified	26 (0.89%)

(B)

No Social Vulnerability (N = 221,152)		Social Vulnerability (N = 12,047)	
**Diagnosis**	**Weighted Frequency (%)**	**Diagnosis**	**Weighted Frequency (%)**
Sepsis, unspecified organism	22476 (10.17%)	Sepsis, unspecified organism	179 (7.02%)
Urinary tract infection, site not specified	6455 (2.92%)	Urinary tract infection, site not specified	50 (1.98%)
Acute kidney failure, unspecified	5398 (2.44%)	Infection following a procedure	43 (1.68%)
Pneumonia, unspecified organism	5197 (2.35%)	Hypertensive heart disease with heart failure	47 (1.84%)
Hypertensive heart and chronic kidney disease with heart failure and stage 1 through stage 4 chronic kidney disease, or unspecified chronic kidney disease	4150 (1.88%)	Acute kidney failure, unspecified	39 (1.52%)
Hypertensive heart disease with heart failure	3881 (1.76%)	Pneumonia, unspecified organism	43 (1.68%)
Pneumonitis due to inhalation of food and vomit	3595 (1.63%)	Alcohol dependence with withdrawal, unspecified	25 (0.98%)
Dislocation of internal left hip prosthesis, initial encounter	3215 (1.45%)	Displaced intertrochanteric fracture of left femur, initial encounter for closed fracture	27 (1.07%)
Other pulmonary embolism without acute cor pulmonale	2929 (1.33%)	Cellulitis of left lower limb	28 (1.11%)
Dislocation of internal right hip prosthesis, initial encounter	2717 (1.23%)	Hypertensive heart and chronic kidney disease with heart failure and stage 1 through stage 4 chronic kidney disease, or unspecified chronic kidney disease	29 (1.13%)
